# Prognostic value of pre-treatment Naples prognostic score (NPS) in patients with osteosarcoma

**DOI:** 10.1186/s12957-020-1789-z

**Published:** 2020-01-30

**Authors:** Qiankun Yang, Tong Chen, Zhongxiang Yao, Xiaojing Zhang

**Affiliations:** 1 Department Bone and Soft Tissue Surgery, Cancer Hospital of China Medical University, Liaoning Cancer Hospital & Institute, Shenyang, China; 2 Department of Physiology, Army Medical University, Chongqing, China

**Keywords:** Osteosarcoma, Prognosis, Survival, NPS, Nomogram

## Abstract

**Background:**

This study aimed to evaluate the clinical significance of pre-treatment Naples prognostic score (NPS) in patients with osteosarcoma.

**Methods:**

The clinical data of 133 osteosarcoma patients between January 2011 and February 2018 in our hospital was retrospectively collected and analyzed. NPS was calculated from four parameters, including serum albumin level, serum total cholesterol (TC), lymphocyte-to-monocyte ratio (LMR), and neutrophil-to-lymphocyte ratio (NLR). Patients were divided into three groups (group 1-3) based on NPS. The relationships between NPS and clinical features, overall survival (OS), and progression-free survival (PFS) were analyzed. Two prediction models based on NPS and clinical parameters were developed: clinical parameters model (model A), and the combined model of NPS and clinical parameters (model B). Their predictive performances were further evaluated and compared.

**Results:**

The median follow-up time of this cohort was 46.0 (range, 5–75) months, while the median OS and PFS was 40 (range, 5–75) months and 36 (range, 5–71) months, respectively. NPS was significantly correlated with gender, tumor location, Enneking stage, pathological fracture, local recurrence, and metastasis (all *P* < 0.05). Variables of NPS, Enneking stage, local recurrence, metastasis, and NLR were confirmed as independent prognostic factors for OS and PFS by univariate and multivariate Cox analysis. Prediction model B obtained larger AUCs for OS and PFS and showed better consistency between nomogram-predicted and actual survival than that of model A at the follow-up time of 1-, 3-, and 5-year.

**Conclusions:**

NPS was a novel, reliable, and multidimensional prognostic scoring system with favorable predictive performance for patients with osteosarcoma.

## Background

Osteosarcoma is the most common malignant bone tumor which predominantly affects adolescents and young adults, accounting for almost 45% of all bone sarcomas [1]. Before the occurrence of multi-disciplinary treatment, the 5-year overall survival (OS) rate was only 10% [2]. With combined modality treatment, namely surgery plus chemotherapy, targeted therapy, or immunotherapy, the 5-year OS significantly increases to 50–70% [3]. A series of factors have been reported in literatures to have predictive or prognostic values for osteosarcoma, including the traditionally established prognostic factors and the newly identified predictive factors. The conventionally established prognostic factors for osteosarcoma included C-reactive protein (CRP), Enneking stage, tumor size, metastasis, alkaline phosphatase, lactate dehydrogenase, pathological fractures, etc. [4–9]. In contrast, some newly reported proteins, micro-RNAs (miRNAs), long non-coding RNAs (lncRNAs), and circular RNAs (circRNAs), such as osteopontin, microRNA-138-5p, lncRNA X-inactive specific transcript (XIST), and circRNA-NT5C2, have also revealed their prognostic significance in osteosarcoma [10–13]. However, all these factors usually covered only one aspect of clinical or pathological characteristics of osteosarcoma patients and thus might be inherently inaccurate and inadequate for prognostic prediction. Furthermore, the high expenses and inconveniences in the detection of these newly identified prognostic biomarkers have restricted its further utilization in routine clinical practice. Consequently, developing a novel, comprehensive, and multidimensional prognostic index composed of easily assessed and easily accessible prognostic factors is a possible way to address this problem.

There is growing evidence that cancer-related inflammation plays crucial roles in the process of tumorigenesis and progression in various malignant tumors, mainly via enhancing angiogenesis and metastasis, suppressing adaptive immune responses, and reducing reactions to chemotherapeutic drugs [14, 15]. High expression of inflammation-related enzymes, proteins, or chemokine receptors in osteosarcoma has been already verified by various studies to correlate with poor outcomes, such as cyclooxygenase-2 (COX-2), matrix metalloproteinases (MMPs), heat shock proteins (HSPs), and chemokine (C-X-C motif) receptor 4 (CXCR4) [16–20]. In addition, the administration of anti-inflammatory drugs during chemotherapy has been confirmed to prolong patients’ survival [21]. Due to the multiple roles of inflammation in osteosarcoma, a series of inflammation-based biomarkers and hematological indices were recommended as prognostic or predictive biomarkers, including CRP, Glasgow prognostic score (GPS), modified Glasgow prognostic score (mGPS), neutrophil-to-lymphocyte ratio (NLR), platelet-to-lymphocyte ratio (PLR), neutrophil-to-platelet score (NPS), mean platelet volume-to-plateletcrit ratio (MPV/PCT), etc. [22–26]. Besides, other prognostic factors which represent or reflect patients’ nutritional or immune status were also confirmed by various studies to be pivotal predictors for survival in osteosarcoma, such as prognostic nutritional index (PNI), the controlling nutritional status (CONUT) score, lymphocyte-to-monocyte (LMR) ratio, systemic immune-inflammation index (SII), etc. [27–30]. Similarly and unfortunately, these predictors also remained somewhat deficient for their limited representation and reflection of patients’ whole status. Therefore, multidimensional prognostic evaluating systems which incorporate multiple prognostic factors together may be better than predictors based on single prognostic factor. Recently, a comprehensive prognostic score, the Naples prognostic score (NPS), calculated from serum albumin and total cholesterol concentrations, LMR and NLR, was reported to be a powerful prognostic index for colorectal cancer (CRC) [31]. NPS is a comprehensive scoring system which includes all of the markers that have been predominantly used now. The prognostic performance of NPS has been validated by a clinical trial (ClinicalTrials.gov Identifier: NCT03272646) with an enrollment of 477 CRC participants and turned out to be the best among all previously reported scoring systems, almost equivalent to the tumor-node-metastasis (TNM) staging system. However, osteosarcoma and CRC are totally two distinct malignancies which differ in multiple aspects, including age of onset, tissue origin, biological behavior, and metastatic site. Therefore, whether NPS has similar prognostic values in osteosarcoma patients remains uncertain. Here, we hypothesized that NPS would obtain optimal prognostic performances in osteosarcoma patients. The aim of our study was to investigate the association between NPS and clinical characteristics, overall survival, and progression-free survival (PFS) in patients with osteosarcoma.

## Methods

### Patient selection

The medical data of 133 osteosarcoma patients from January 2011 to February 2018 in Cancer Hospital of China Medical University (also known as Liaoning Cancer Hospital & Institute) was collected and coded for further analysis. The inclusion criteria for this study were as follows: (i) patients were pathologically diagnosed with osteosarcoma, (ii) patients received no prior anti-cancer treatment, (iii) patients with detailed and extractable medical data and laboratory results, and (iv) patients were not lost to follow-up. Participants who meet either of the following criteria were excluded from the final analysis: (i) patients have any clinical evidences of infection or inflammatory diseases. In this study, infection was defined as a condition of body temperature over ≥ 37.5 °C (99.5 °F) and with positive outcomes from peripheral blood microbial culture. Inflammatory diseases refer to a large set of disorders characterized by systemic and organ-specific inflammation, as well as an elevated level of CRP, procalcitonin, and erythrocyte sedimentation rate (ESR) [32, 33]. (ii) Patients have history of other malignancies, or they have been previously treated with any anti-cancer agents, non-steroid anti-inflammatory drugs (NSAIDs), or antibiotics. An infectious or inflammatory status, or the administration of specific agents to intervene such conditions would affect the accuracy of blood test, so patients with any record of these conditions mentioned above were excluded from this study [34–37]. (iii) Patients with incomplete medical records or laboratory results were also excluded. This study was approved by the medical ethics committee of Liaoning Cancer Hospital & Institute. The data are anonymous, and therefore the requirement for informed consent was waived.

### Data collection and NPS definition

The following clinical features and pathological parameters of patients were retrospectively collected from the hospital information system, including gender, age, tumor location, tumor size, histological type, recurrence, Enneking stage, pathological fracture, metastasis status, neoadjuvant chemotherapy, and laboratory data. The data of neutrophil, lymphocyte, monocyte, and platelet was obtained from regular blood test, and the serum albumin and plasma cholesterol levels came from hepatic function test, with the blood sample taken for examination before breakfast in the morning. The NLR and LMR derived from routine blood test were calculated as total neutrophil count divided by total lymphocyte count and total neutrophil count divided by total monocyte count, respectively. The definition of NPS was based on the following four parameters, namely serum albumin, total cholesterol (TC), LMR, and NLR. As previously reported by Gennaro Galizia et al. [31], the cut-off values were 4 mg/dL for serum albumin, 180 mg/dL for TC, 2.96 for NLR, and 4.44 for LMR, respectively. Patients with serum albumin, TC or LMR lower than 4 mg/dL, 180 mg/dL, and 4.44 got one point; otherwise, they got zero point. As for NLR, patients with NLR higher than 2.96 got one point, while those with NLR lower than 2.96 got zero point. The sum of the score from each parameter was NPS. Patients were categorized into three groups according to NPS: patients with NPS of 0 were assigned to group 1, patients with NPS of 1 or 2 were defined as group 2, and patients with NPS of 3 or 4 were considered as group 3 (Table 1).

**Table 1:** Calculation of Naples prognostic score (NPS)

Variables	Cut-off value	Points	NPS group
Serum albumin (mg/dL)	≥ 4	0	Group 1: 0 point Group 2: 1 or 2 points Group 3: 3 or 4 points
	< 4	1	
TC (mg/dL)	> 180	0	
	≤ 180	1	
NLR	≤ 2.96	0	
	> 2.96	1	
LMR	> 4.44	0	
	≤ 4.44	1	

*TC* total cholesterol, *NLR* neutrophil-to-lymphocyte ratio, *LMR* lymphocyte-to-monocyte ratio, *NPS* Naples prognostic score

### Follow-up

All patients were regularly followed up after the initiation of treatment (adjuvant chemotherapy or surgery). Patients were contacted mainly via outpatient examination or phone call according to our institutional rules and regulations. The time intervals for follow-up were every 3 months for the first 3 years, and then every 6 months in the following years. Physical examination, laboratory test, chest radiography, as well as radiograph of the primary lesion locations were routinely performed. Patients were followed up until death or February 2018. The OS was defined as the time period from the first treatment to death (event) or the last follow-up (censored), and the PFS was calculated from initiation of therapy to disease progression, usually including metastasis, recurrence, or death.

### Statistical analyses

The IBM SPSS Statistics 24 (SPSS, Inc., Chicago, IL) and R software (version 3.6.0) were utilized to perform all statistical analyses. The associations between categorical variables were analyzed with chi-square test or Fisher’s exact test. The receiver operating characteristic (ROC) curve analysis was used to identify the predictive accuracy of NPS and its constituent parameters. The Kaplan-Meier method and Log-rank test were utilized to compare the differences in survival among NPS groups. Prognostic factors were analyzed and selected by univariate and multivariate Cox proportional hazards regression analyses. Hazard ratios (HRs) and their 95% confidence intervals (CIs) of all variables were also calculated. Two predictive models to predict median survival time (MST), and the probabilities of 3- and 5-year OS and PFS were constructed based on univariate and multivariate Cox analyses. Time-dependent ROC curve analyses were performed to compare the predicting efficiency of the two prediction models. Calibration curves were plotted to evaluate the consistency between predicted and observed survival. A two-tailed *P* value lower than 0.05 was deemed as statistically significant.

## Results

### Patient characteristics

A total of 133 osteosarcoma participants were enrolled in this study according to the inclusion and exclusion criteria. Among them, 74 (55.64%) were males and 59 (44.36%) were females, with the median age of 18 (range, 5–68) years. With regard to the tumor site, 108 (81.20%) tumors were primarily located in extremities and 25 (18.80%) tumors primarily occurred in non-extremities. Eighty-six (64.66%) patients had tumor size smaller than 10.5 cm, and 47 (35.34%) patients had tumor size larger than 10.5 cm, with the median tumor size of 10.5 (range, 1.2–19.5) cm. Besides, 107 (80.45%) patients and 26 (19.55%) patients had well-differentiated and poorly differentiated histology subtypes, respectively. Other variables, such as Enneking stage, pathological fractures, local recurrence, metastasis, and neoadjuvant chemotherapy, were presented in Table 3.

### The cut-off values, AUC, sensitivity, and specificity for NPS and its constituent parameters

**Fig. 1:**
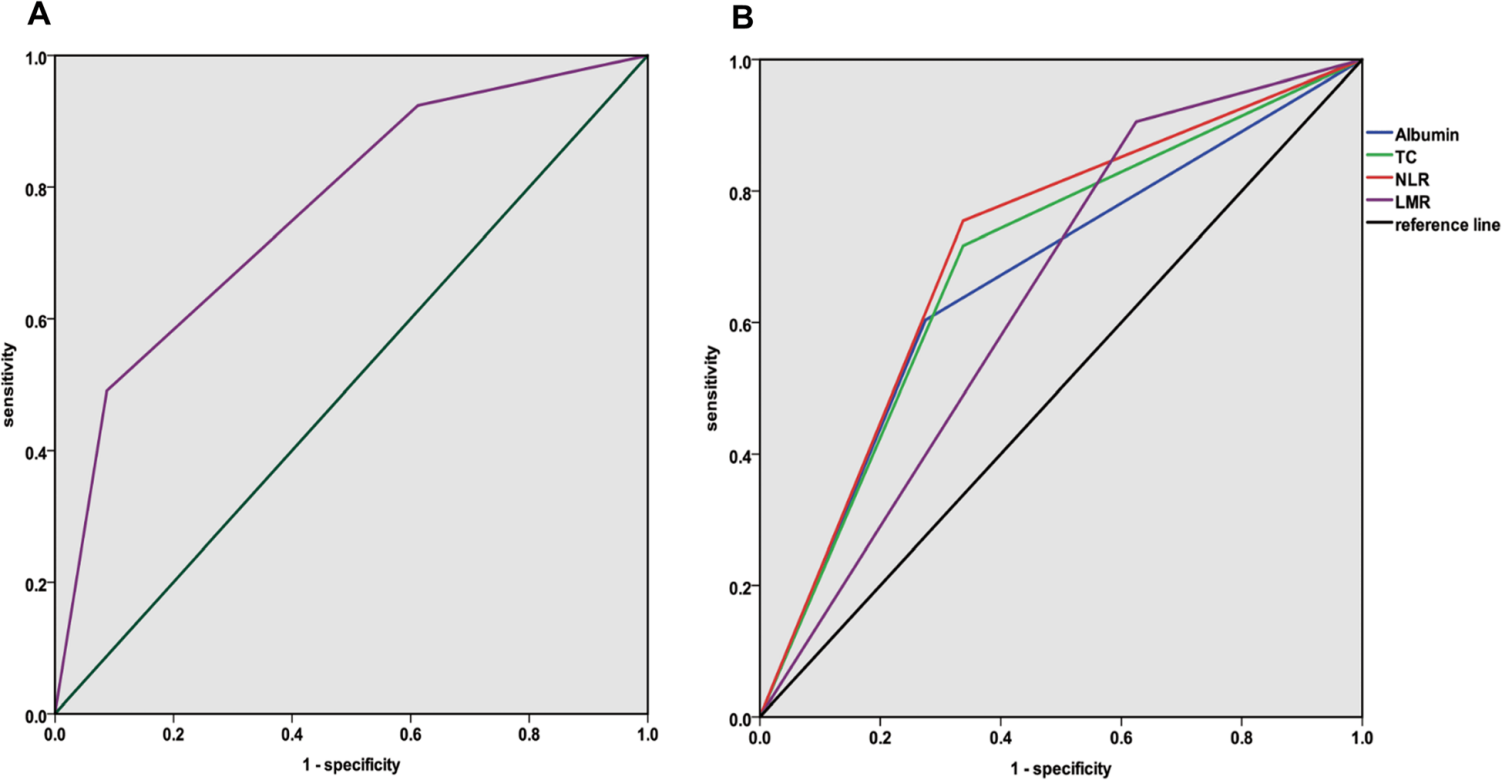
The ROC curve analyses for pre-treatment **a** NPS and **b** variables of NLR, LMR, TC, and albumin. *ROC* receiver operating characteristic, *NPS* Naples prognostic score, *NLR* neutrophil-to-lymphocyte ratio, *LMR* lymphocyte-to-monocyte ratio, *TC* total cholesterol

Based on the cut-off values presented in Table 1, we evaluated the diagnostic performance of these indices by using ROC curve analysis. The area under the curve (AUC), sensitivity, and specificity for NPS and its constituent parameters were shown in Table 2, and the ROC curves for NPS and its constituent parameters were presented in Fig. 1. NPS got the largest AUC (0.766), sensitivity (90.9%), and specificity (86.4%) compared with its constituent variables.

**Table 2:** Cut-off values and AUC for NPS and its constituent parameters

Prognostic system	Cut-off value	AUC	Sen (%)	Spe (%)
Albumin (mg/dL)	4	0.664	60.2	74.3
TC (mg/dL)	180	0.690	70.1	65.2
NLR	2.96	0.709	78.3	72.5
LMR	4.44	0.640	90.3	40.6
NPS^a^	-	0.766	90.9	86.4

*NPS* Naples prognostic score, *NLR* neutrophil-to-lymphocyte ratio, *LMR* lymphocyte-to-monocyte ratio, *AUC* area under the curve, *Sen* sensitivity, *Spe* specificity

^a^NPS is a categorical variable. The cut-off values of these prognostic systems were determined by reference but not by ROC curve analysis. (Gennaro G et. al. 2017, [31])

### Association between NPS and clinicopathological characteristics

The baseline characteristics of osteosarcoma patients based on NPS group were shown in Table 3. NPS was significantly associated with gender (*P* < 0.001), tumor location (*P* = 0.009), Enneking stage (*P* < 0.001), pathological fracture (*P* = 0.003), local recurrence (*P* < 0.001), and metastasis (*P* = 0.002). Distribution of age, tumor size, histological type, and neoadjuvant chemotherapy did not differ significantly among NPS groups.

**Table 3:** Baseline characteristics of patients based on NPS group

Variables	Total cases (*N* = 133)	Group 1 (*n* = 35)	Group 2 (*n* = 65)	Group 3 (*n* = 33)	*P* value
Gender					0.000
Male	74 (55.64%)	29 (82.86%)	34 (52.31%)	11 (33.30%)	
Female	59 (44.36%)	6 (17.14%)	31 (47.69%)	22 (66.70%)	
Age					0.432
< 18 years	41 (30.83%)	8 (22.86%)	23 (35.38%)	10 (30.30%)	
≥ 18 years	92 (69.17%)	27 (77.14%)	42 (64.62%)	23 (69.70%)	
Tumor location					0.009
Extremities	108 (81.20%)	33 (94.28%)	46 (70.77%)	29 (87.87%)	
Non-extremities	25 (18.8%)	2 (5.72%)	19 (29.23%)	4 (12.13%)	
Tumor size					0.257
< 10.5 cm	86 (64.66%)	20 (57.14%)	41 (63.08%)	25 (75.76%)	
≥ 10.5 cm	47 (35.34%)	15 (42.86%)	24 (36.92%)	8 (24.24%)	
Histological type					0.070
Well-differentiated	107 (80.45%)	30 (85.71%)	55 (84.62%)	22 (66.67%)	
Poorly differentiated	26 (19.55%)	5 (14.29%)	10 (15.38%)	11 (33.33%)	
Enneking stage					0.000
I/II	103 (77.44%)	31 (91.43%)	59 (90.77%)	13 (39.40%)	
III	30 (22.56%)	4 (8.57%)	6 (9.23%)	20 (60.60%)	
Pathological fracture					0.005^a^
No	123 (92.48%)	34 (97.14%)	63 (96.23%)	26 (78.79%)	
Yes	10 (7.52%)	1 (2.86%)	2 (3.77%)	7 (21.21%)	
Local recurrence					0.000^a^
No	119 (89.47%)	35 (100%)	64 (98.46%)	20 (60.60%)	
Yes	14 (10.53%)	0 (0%)	1 (1.54%)	13 (39.40%)	
Metastasis					0.003^a^
No	123 (92.48%)	35 (100%)	62 (95.38%)	26 (78.79%)	
Yes	10 (7.52%)	0 (0%)	3 (4.62%)	7 (21.21%)	
Neoadjuvant CT					0.228
No	93 (69.92%)	23 (65.71%)	43 (66.15%)	27 (81.82%)	
Yes	40 (30.08%)	12 (34.29%)	22 (33.85%)	6 (18.18%)	

*NPS* Naples prognostic score, *CT* chemotherapy

^a^Fisher’s exact test

### Prognostic factors and their predictive performances for OS and PFS

The median follow-up time of this retrospective cohort was 46.0 (range, 5–75) months. The median OS and PFS was 40 (range, 5–75) months and 36 (range, 5–71) months, respectively.

**Fig. 2:**
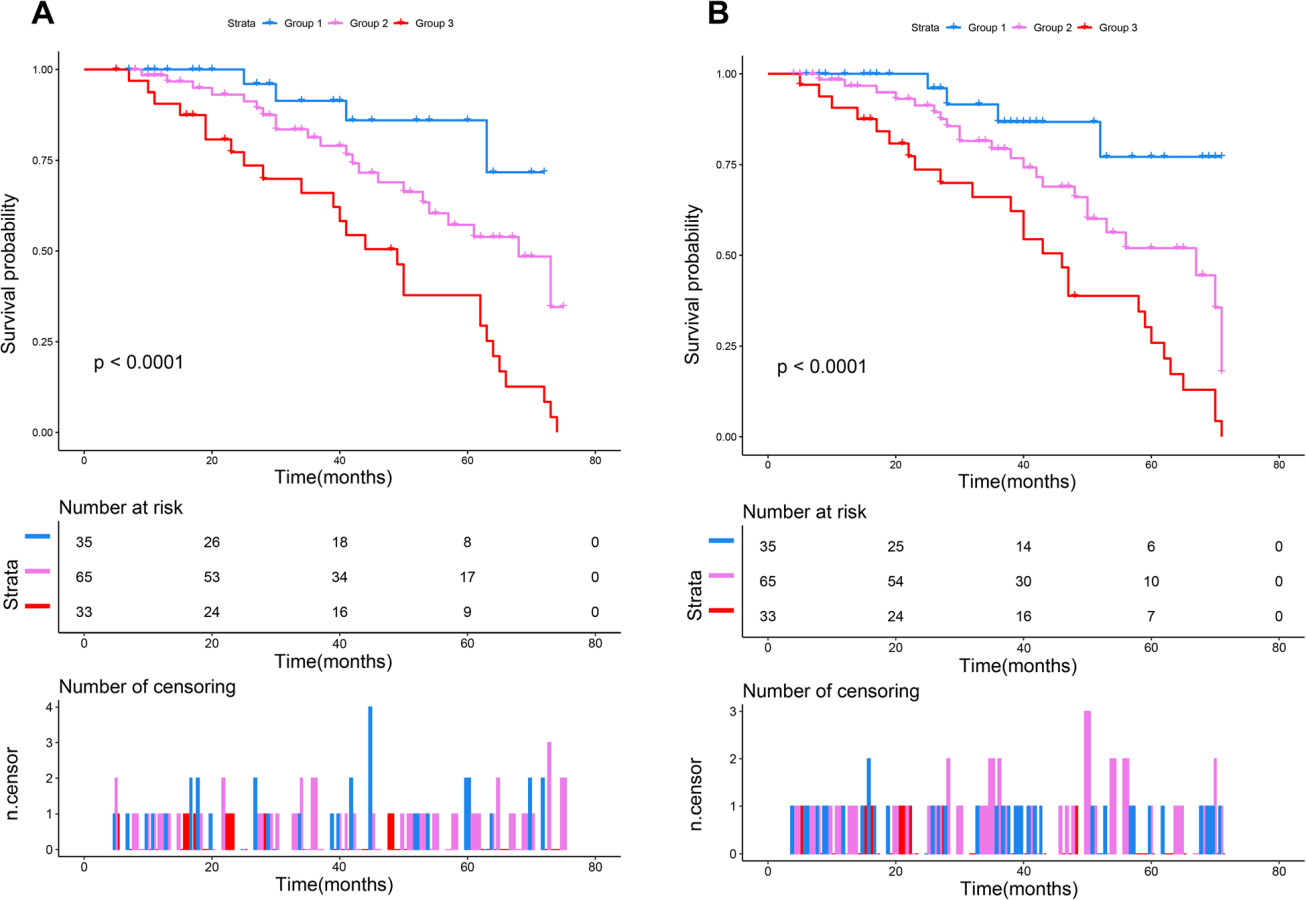
Kaplan-Meier survival curves for **a** OS and **b** PFS based on NPS stratification. *OS* overall survival, *PFS* progression-free survival, *NPS* Naples prognostic score

The univariate and multivariate Cox analyses for OS and PFS were presented in Tables 4 and 5, respectively. In univariate analysis, OS was significantly related to tumor size (*P* = 0.026), Enneking stage (*P* < 0.001), pathological fractures (*P* = 0.039), local recurrence (*P* = 0.033), metastasis (*P* < 0.001), NLR (*P* < 0.001), LMR (*P* = 0.004), albumin (*P* = 0.037), and NPS (*P* < 0.001) (Table 4). Except for pathological fractures, all the variables mentioned above were also significantly associated with PFS (Table 5). Compared with group 1, patients in groups 2 and 3 had worse OS [NPS group 2 vs. group 1, HR = 4.323 (95% CI 0.996–9.852), *P* < 0.001; NPS group 3 vs. group 1: HR = 7.073 (95% CI 1.188–15.124), *P* < 0.001]. Similarly, patients in groups 2 and 3 also had poorer PFS compared with patients in group 1 [NPS group 2 vs. group 1: HR = 5.672 (95% CI 1.254–10.003), *P* < 0.001; NPS group 3 vs. group 1: HR = 7.841 (95% CI 1.029–11.565), *P* < 0.001]. The Kaplan-Meier survival analyses based on NPS stratification also confirmed this finding (Fig. 2). The log-rank Chi-squared statistics for trend were 15.108 (*P* < 0.001) and 13.532 (*P* = 0.001) for OS and PFS, respectively. In multivariate analysis, Enneking stage (*P* < 0.001), local recurrence (*P* < 0.001), metastasis (*P* < 0.001), NLR (*P* < 0.001), and NPS (*P* < 0.001) were confirmed to be independent prognostic factors for OS (Table 4) and PFS (Table 5).

**Table 4:** Univariate and multivariate Cox analysis for OS

Variables	Univariate analysis		Multivariate analysis	
	HR (95% CI)	*P* value	HR (95% CI)	*P* value
Gender		0.872		0.693
Male	Reference		Reference	
Female	0.941 (0.47–1.69)		1.013 (0.341–1.592)	
Age (years)		0.443		0.565
< 18	Reference		Reference	
≥ 18	1.152 (0.957–1.344)		1.094 (0.982–1.415)	
Tumor location		0.465		0.621
Extremities	Reference		Reference	
Non-extremities	1.123 (0.892–1.458)		1.075 (0.917–1.273)	
Tumor size (cm)		0.026		0.373
< 10.5	Reference		Reference	
≥ 10.5	2.378 (1.087–3.346)		1.426 (0.719–2.765)	
Histological type		0.052		0.124
Well-differentiated	Reference		Reference	
Poorly differentiated	1.463 (1.275–2.897)		1.205 (0.894–2.319)	
Enneking stage		< 0.001		< 0.001
I/II	Reference		Reference	
III	3.140 (1.021–7.361)		3.820 (0.172–8.909)	
Pathological fracture		0.039		0.318
No	Reference		Reference	
Yes	1.222 (1.077–8.418)		1.219 (0.893–7.029)	
Local recurrence		0.033		< 0.001
No	Reference		Reference	
Yes	3.563 (1.359–6.379)		4.162 (1.085–9.153)	
Metastasis		< 0.001		< 0.001
No	Reference		Reference	
Yes	5.378 (1.359–6.379)		6.482 (1.985–13.647)	
Neoadjuvant CT		0.447		0.624
No	Reference		Reference	
Yes	0.897 (0.296–1.854)		0.845 (0.404–1.532)	
NLR		< 0.001		< 0.001
Low (≤ 2.96)	Reference		Reference	
High (> 2.96)	3.197 (1.786–6.454)		3.986 (0.781–6.239)	
LMR		0.004		0.057
Low (≤ 4.44)	Reference		Reference	
High (> 4.44)	0.786 (0.457–2.345)		0.855 (0.753–1.783)	
Albumin		0.037		0.126
Low (< 4 mg/dL)	Reference		Reference	
High (≥ 4 mg/dL)	0.887 (0.768–1.508)		0.975 (0.684–1.651)	
TC		0.782		0.651
Low (≤ 180 mg/dL)	Reference		Reference	
High (> 180 mg/dL)	0.898 (0.654–1.317)		1.056 (0.737–2.249)	
NPS Group				
1	Reference		Reference	
2	4.323 (0.996–9.852)	< 0.001	5.873(1.031–6.428)	< 0.001
3	7.073 (1.188–15.124)	< 0.001	6.547(1.153–13.624)	< 0.001

*CI* confidence interval, *CT* chemotherapy, *NLR* neutrophil-to-lymphocyte ratio, *LMR* lymphocyte-to-monocyte ratio, *TC* total cholesterol, *NPS* Naples prognostic score, *OS* overall survival

**Table 5:** Univariate and multivariate Cox analysis for PFS

Variables	Univariate analysis		Multivariate analysis	
	HR (95% CI)	*P* value	HR (95% CI)	*P* value
Gender		0.785		0.883
Male	Reference		Reference	
Female	0.879 (0.568–1.457)		1.102 (0.487–1.569)	
Age (years)		0.415		0.845
< 18	Reference		Reference	
≥ 18	1.254 (0.742–2.124)		1.126 (0.974–1.951)	
Tumor location		0.576		0.451
Extremities	Reference		1	
Non-extremities	1.375 (0.825–2.273)		1.255 (0.998–2.989)	
Tumor size (cm)		0.021		0.165
< 10.5	Reference		Reference	
≥ 10.5	2.147 (1.126–3.589)		1.362 (1.079–4.532)	
Histological type		0.457		0.752
Well-differentiated	Reference		Reference	
Poorly differentiated	1.541 (0.856–3.124)		1.336 (1.0719–3.257)	
Enneking stage		< 0.001		< 0.001
I/II	Reference		Reference	
III	4.577 (1.036–12.539)		6.457 (1.324–10.987)	
Pathological fracture		0.341		0.542
No	Reference		Reference	
Yes	1.243 (0.756–3.493)		1.322 (0.893–5.786)	
Local recurrence		< 0.001		< 0.001
No	Reference		Reference	
Yes	5.467 (1.095–9.852)		6.991 (2.048–11.548)	
Metastasis		< 0.001		< 0.001
No	Reference		Reference	
Yes	6.678 (3.589–10.679)		7.895 (4.470–12.907)	
Neoadjuvant CT		0.688		0.785
No	Reference		Reference	
Yes	0.842 (0.413–1.759)		0.903 (0.378–2.235)	
NLR		< 0.001		< 0.001
Low (≤ 2.96)	Reference		Reference	
High (> 2.96)	4.517 (1.657–8.689)		4.652 (1.329–9.547)	
LMR		0.003		0.062
Low (≤ 4.44)	Reference		Reference	
High (> 4.44)	0.751 (0.556–1.579)		0.876 (0.657–1.322)	
Albumin		0.046		0.257
Low (< 4 mg/dL)	Reference		Reference	
High (≥ 4 mg/dL)	0.854 (0.687–2.211)		0.859 (0.789–1.457)	
TC		0.142		0.457
Low (≤ 180 mg/dL)	Reference		Reference	
High (> 180 mg/dL)	0.984 (0.568–1.788)		1.054 (0.891–2.014)	
NPS Group				
1	Reference		Reference	
2	5.672 (1.254–10.003)	< 0.001	5.272 (1.017–11.485)	< 0.001
3	7.841 (1.029–11.565)	< 0.001	6.783 (1.234–10.575)	< 0.001

*CI* confidence interval, *CT* chemotherapy, *NLR* neutrophil-to-lymphocyte ratio, *LMR* lymphocyte-to-monocyte ratio, *TC* total cholesterol, *NPS* Naples prognostic score, *PFS* progression-free survival

**Fig. 3:**
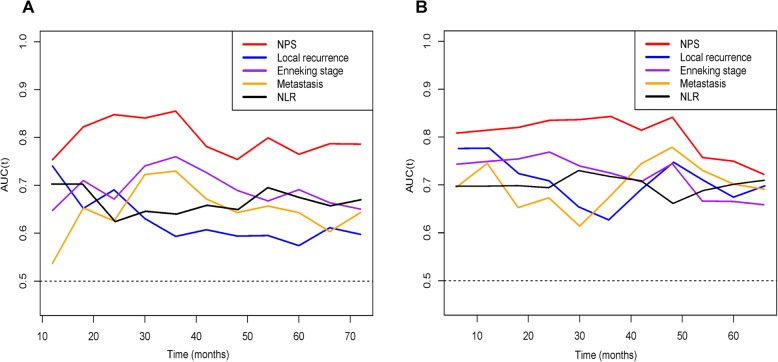
The time-dependent AUC curve analyses of prediction models for **a** OS and **b** PFS. The *X*-axis symbolizes the follow-up time, and the *Y*-axis represents estimated AUC for survival at specific time of interest. *OS* overall survival, *PFS* progression-free survival, *NPS* Naples prognostic score, *NLR* neutrophil-to-lymphocyte ratio

The time-dependent ROC curve analyses were performed to compare the predictive performances among different independent prognostic factors. NPS obtained the highest AUCs in dynamic trends among all variables within the follow-up time (Fig. 3).

### Construction and evaluation of prediction models based on NPS and clinical parameters

**Fig. 4:**
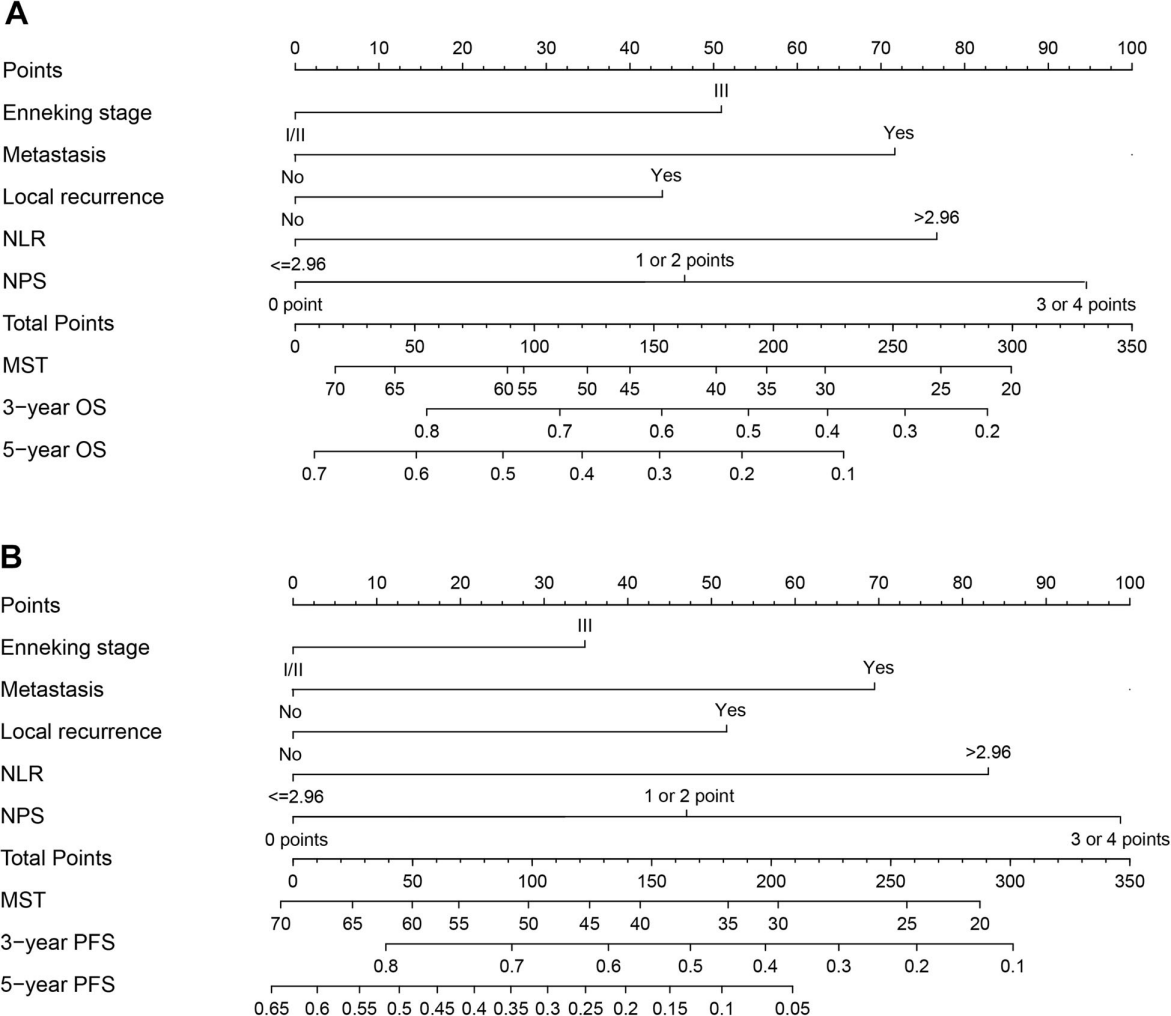
Nomograms based on NPS and clinical prognostic factors to predict MST and the probabilities of 3- and 5-year **a** OS and **b** PFS. *MST* median survival time, *OS* overall survival, *PFS* progression-free survival, *NPS* Naples prognostic score

**Fig. 5:**
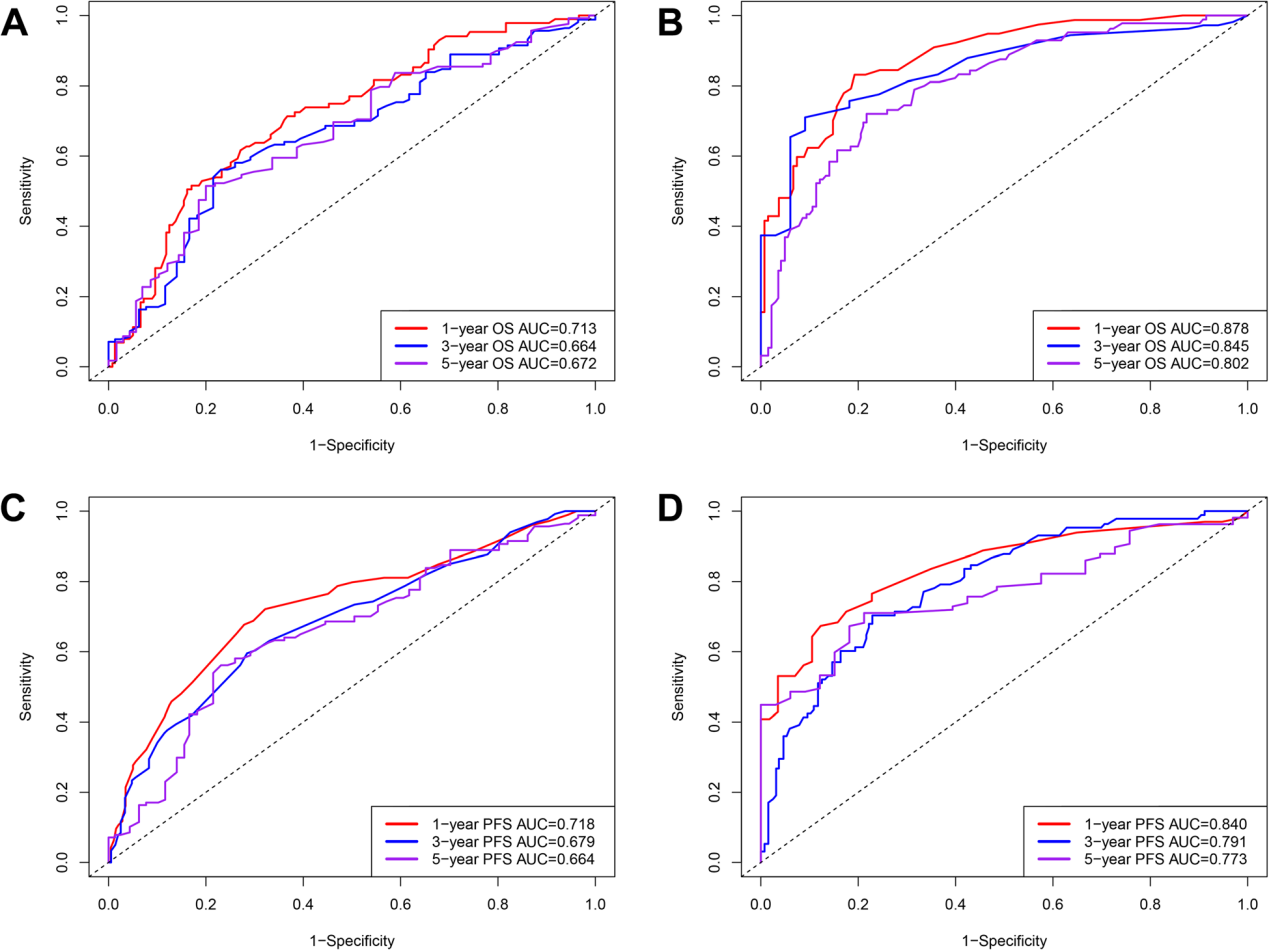
The time-dependent ROC curve analyses of prediction models for 1-, 3-, and 5-year survival. The ROC curves of clinical parameters model for 1-, 3-, and 5-year **a** OS and **c** PFS. The ROC curves of combined model of NPS and clinical parameters for 1-, 3-, and 5-year **b** OS and **d** PFS. *AUC* area under the curve, *ROC* receiver operating characteristic, *OS* overall survival, *PFS* progression-free survival

**Fig. 6:**
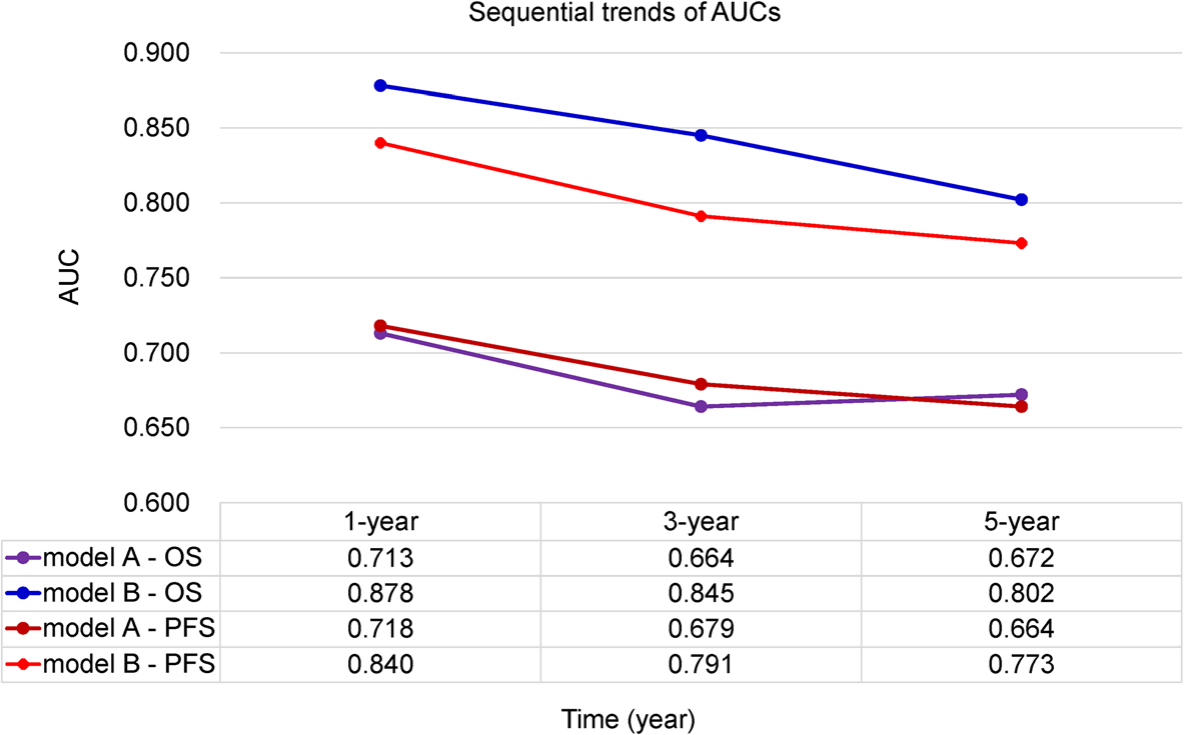
Line chart illustrating the sequential trends in AUC between the two prediction models at 1-, 3-, and 5-year survival. The horizontal axis symbolizes the survival time, and the vertical axis represents estimated AUC for survival at specific time of interest. *AUC* area under the curve, *ROC* receiver operating characteristic, *OS* overall survival, *PFS* progression-free survival

**Fig. 7:**
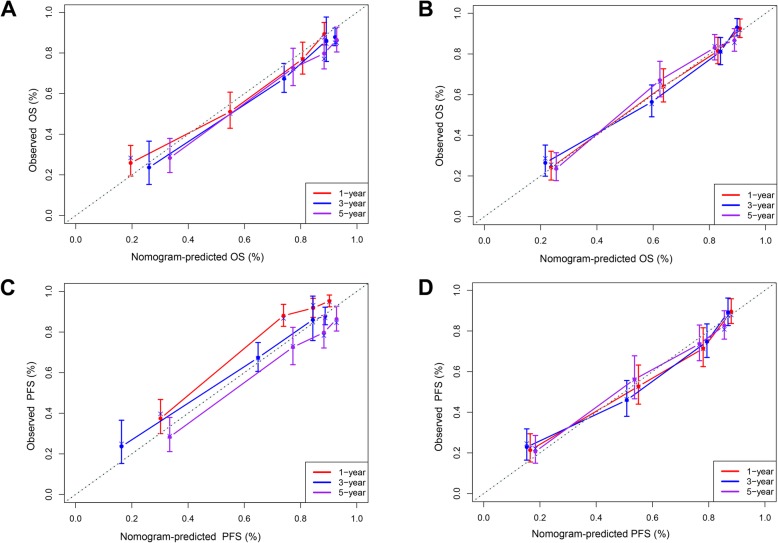
Calibration curves of prediction models for predicting the 1-, 3-, and 5-year survival. The clinical parameters model for predicting 1-, 3-, and 5-year **a** OS and **c** PFS. The combined model of NPS and clinical parameters for predicting 1-, 3-, and 5-year **b** OS and **d** PFS. The horizontal axis represents the nomogram-predicted survival, and the vertical axis symbolizes the actual survival. The curve in color closest to the 45° gray dotted line gets the best prediction performance. *OS* overall survival, *PFS* progression-free survival

In order to further confirm the clinical significance of NPS in this cohort, two prognostic models to predict MST, and the probabilities 3- and 5-year survival were constructed: clinical parameters model (model A) and the combined model (model B) of NPS and clinical parameters. Variables of Enneking stage, metastasis, local recurrence, and NLR were included in model A while NPS and the above-mentioned variables were included in model B. The nomograms of model B for predicting MST, OS, and PFS were shown in Fig. 4. The performances of the established two models were further evaluated and compared by performing the time-dependent ROC curve analysis and plotting the calibration curves. The time-dependent ROC curves of model A and model B for predicting 1-, 3-, and 5-year OS and PFS were presented in Fig. 5 and sequential trends in AUCs were illustrated in Fig. 6. Compared with model A, model B got larger AUCs for OS and PFS at the follow-up time of 1-, 3-, and 5-year. The calibration curves of model A and model B for predicting 1-, 3-, and 5-year OS and PFS were shown in Fig. 7. Model B showed better agreement between predicted survival and actual survival at the survival time of 1-, 3-, and 5-year.

## Discussion

Our study indicates that NPS is an independent prognostic indicator for the outcomes of patients with osteosarcoma. Patients in NPS group 2/3 are more prone to get worse OS and PFS compared with those in NPS group 1. Furthermore, NPS shows better prognostic performances than its parameters, with a lager AUC of 0.766 and relatively higher sensitivity of 90.9% and specificity of 86.4%, respectively. Similarly, the combined model of NPS and clinical parameters also obtains higher discriminatory ability and shows better consistency between predicted survival and actual survival for 1-, 3-, and 5-year OS and PFS. Most importantly, all easily assessed and predominantly widely used variables were integrated together by NPS, making it a more representative and reflective predictor for osteosarcoma.

It is suggested that malnutrition is closely associated with carcinogenesis, cancer growth, and tumor progression, including osteosarcoma, leading to the search and formulation for biomarkers or prognostic scoring systems based on nutrition [30, 38]. Malnutrition correlates with unfavorable prognosis in a variety of tumors [39, 40]. In particular, hypoalbuminemia is not only a marker for malnutrition but also serves as an indicator for systemic inflammation, because the concentration of albumin can be exhausted by some pro-inflammatory substances, such as cytokines. When precluding the influences from other interfering factors, a low level of serum albumin in a patient usually represents a status of a high inflammation or a disease of high malignancy. Given its crucial significance in malignancies, serum albumin levels are covered by almost all nutritional prognostic scoring system, such as GPS, mGPS, Hs-mGPS, PNI, C-reactive protein to albumin ratio (CAR), etc. [24, 27, 41, 42]. However, a limitation of albumin concentrations is that it can be easily affected by liver function and changes of body fluid volume [43], so some authors have proposed to take plasma total cholesterol levels into account to optimize the nutritional evaluation system [44, 45]. Cholesterol, integrated into specialized lipid-protein membrane micro-domains, forms the signal transduction machinery and is involved in key cellular signaling pathways that are responsible for malignant transformation via modulation of cytoskeleton alteration, cell polarity, and angiogenesis [46–50]. Hypocholesterolemia has been reported to correlate with worse outcomes and prognosis in a variety of tumors, such as renal cell carcinoma (RCC), CRC, breast cancer, etc. [51–53]. Low levels of cholesterol can influence cell membrane fluidity, inhibiting the mobility of cell surface receptors and finally interfering their ability for transmembrane signals transmission [54]. When it happens in immune cells, previous immuno-competent cells may become immuno-incompetent cells and are unable to supervise and destroy cancer cells which often express a small quantity of neoantigens at the initial phase of tumor onset. The coverage of plasma total cholesterol levels and serum albumin levels into NPS might better reflect the nutritional status of patients and enhance performance in prognostic stratification. In our study, serum albumin and TC levels were not independent prognostic factors for the survival of osteosarcoma patients, but they formulate a new scoring system of NPS which showed good prognostic performance when combined with NLR and LMR.

Cancer-related inflammation and cell-mediated immune responses also play vital roles in cancer development and progression, and they are largely dependent on neutrophils, lymphocytes and monocytes. Neutrophilia, monocytosis, and lymphopenia are nonspecific responses to cancer-related inflammation and immune reaction and are related to poor survival in malignancies. Neutrophils can interact with tumor cells via producing cytokines and chemokines, which mainly regulates tumor cells’ proliferation, angiogenesis, and metastasis [55]. Tumor-associated macrophages are differentiated from blood monocytes and also involved in tumor progression and metastases [56]. With regard to lymphocytes, they play a crucial role in cell-mediated immune response by recognizing and destructing cancer cells [57]. Up to now, a growing number of prognostic parameters based on immune cells have been formulated and reported by studies, including NLR, PLR, LMR, and NPS. Elevated NLR, PLR, NPS and decreased LMR in patients were often associated with poor prognosis [25, 58]. Consistent with previous studies, our study also confirmed that high NLR and low LMR were significantly correlated with poor survival. In fact, similar to the changes in peripheral blood immune cells, the variations of immune infiltrations in the tumor microenvironment (TME) are also reliable and effective prognostic factors for many tumors, including bone and soft tissue sarcoma. In osteosarcoma, high ratio of tumor-infiltrating macrophages (TAMs) and CD8^+^ cytotoxic lymphocytes (CTLs) in TME are closely related to favorable prognosis, whereas low ratio of immune infiltrations has been considered as a predictor for poor outcome [59–61]. Moreover, osteosarcoma patients with elevated CD8(+)/FOXP3(+) ratio and CD8^+^/Treg ratio in TME often harvest improved survival [62, 63]. In addition, early peripheral blood lymphocyte recovery after initiation of chemotherapy is a reliable prognostic indicator for superior outcome in patients with osteosarcoma [29, 64, 65]. By incorporating all these important, easily available and widely used biomarkers into NPS, a comprehensive predictive tool which represents a patients’ whole status in multidimensional aspects can be formulated. We further constructed a prediction model based on NPS, and its predictive performance for OS and PFS proved to be much better than that of clinical parameters model, indicating its superiorities over other predictors in pre-treatment prognostic stratification.

This study also has some limitations. Firstly, our study is a retrospective, single-institution study and the sample was relatively small, which may lead to some discrepancies compared with previous studies. For instance, significant differences were found between gender and NPS groups in this study. However, gender was not deemed as a pivotal prognostic indicator for osteosarcoma in previous studies, and this discrepancy may represent a kind of selection bias. Secondly, the cut-off values of albumin, TC, NLR, and LMR came from the references reported in previous studies, but not determined by ROC curve analysis based on the highest Youden’s index, which may pose some influences to the results of our study. Even so, the NPS in our study, with the cut-off values of its parameters unmodified, also presented favorable prognostic performances in predicting survival in osteosarcoma patients. Thirdly, other important predictive biomarkers such as CRP, GPS, mGPS, and ALP were not analyzed in our study. Finally, despite for its advantages in this cohort, it is noteworthy that NPS is a non-specific predictor for osteosarcoma and thus unavoidably possesses its intrinsic weaknesses and limitations. By combining NPS with some specific biomarkers for osteosarcoma, such as miR-138-5p, circ_0000502, lncRNA TP73-AS1, and circ-NT5C2, numerous novel, specific, and multidimensional prognostic indexes can be formulated, but their prognostic performances need to be verified in future studies [10, 12, 66, 67]. So, future studies can concentrate on screening optimal combinations of NPS and new biomarkers for osteosarcoma.

## Conclusions

In summary, we studied the clinical significance and prognostic values of NPS in a cohort of osteosarcoma patients from our institution. A prediction model based on NPS and clinical parameters was established and evaluated, and this model turned out to be more reliable and accurate than prediction model based on clinical parameters only. Therefore, NPS might be a novel and promising inflammation-, immunity-, and nutrition-based comprehensive index for pre-treatment prognostic stratification in patients with osteosarcoma. Early detection and improvement of malnutrition and inflammation, especially for patients in NPS group 3, may lead to amelioration of systemic inflammation and improvement of outcomes.

## Data Availability

The dataset supporting the conclusions of this article is included within the article.

## Abbreviations

AUC: Area under the curve
CXCR4: Chemokine (C-X-C motif) receptor 4
circRNAs: Circular RNAs
CRC: Colorectal cancer
CI: Confidence interval
CONUT: Controlling nutritional status
CRP: C-reactive protein
CAR: C-reactive protein to albumin ratio
COX-2: Cyclooxygenase-2
CTLs: Cytotoxic lymphocytes
GPS: Glasgow prognostic score
HRs: Hazard ratios
HSPs: Heat shock proteins
lncRNA: Long non-coding RNA
MMPs: Matrix metalloproteinases
MPV/PCT: Mean platelet volume-to-plateletcrit
MST: Median survival time
miRNA: Micro-RNAs
mGPS: Modified Glasgow prognostic score
NPS: Naples prognostic score
NLR: Neutrophil-to-lymphocyte ratio
NSAIDs: Non-steroid anti-inflammatory drugs
OS: Overall survival
PLR: Platelet-to-lymphocyte ratio
PNI: Prognostic nutritional index
PFS: Progression-free survival
RCC: Renal cell carcinoma
TC: Total cholesterol
TNM: Tumor-node-metastasis
TME: Tumor microenvironment
TAMs: Tumor-infiltrating macrophages
XIST: X-inactive specific transcript
